# Speciation, Phenotypic Variation and Plasticity: What Can Endocrine Disruptors Tell Us?

**DOI:** 10.1155/2013/862739

**Published:** 2013-05-16

**Authors:** Braulio Ayala-García, Marta López-Santibáñez Guevara, Lluvia I. Marcos-Camacho, Alma L. Fuentes-Farías, Esperanza Meléndez-Herrera, Gabriel Gutiérrez-Ospina

**Affiliations:** ^1^Laboratorio de Ecofisiología Animal, Departamento de Zoología, Instituto de Investigaciones Sobre Recursos Naturales, Universidad Michoacana de San Nicolás de Hidalgo, Morelia, MI, Mexico; ^2^Laboratorio de Biología de Sistemas, Departamento de Biología Celular y Fisiología, Instituto de Investigaciones Biomédicas, Universidad Nacional Autónoma de México, 04510 Ciudad de México, Mexico

## Abstract

Phenotype variability, phenotypic plasticity, and the inheritance of phenotypic traits constitute the fundamental ground of processes such as individuation, individual and species adaptation and ultimately speciation. Even though traditional evolutionary thinking relies on genetic mutations as the main source of intra- and interspecies phenotypic variability, recent studies suggest that the epigenetic modulation of gene transcription and translation, epigenetic memory, and epigenetic inheritance are by far the most frequent reliable sources of transgenerational variability among viable individuals within and across organismal species. Therefore, individuation and speciation should be considered as nonmutational epigenetic phenomena.

## 1. Introduction

 Phenotypic variability among individuals within different organismal species is essential for them to prosper. Indeed, by expanding each species phenotypic repertoire, the possibility of organism populations to overcome environmental contingencies increases. Phenotypic variability is not only important at the species level to improve the chances of assuring their continuity over time; it is also at the heart of the emergence of new species during the process of evolution. For decades, evolutionary thought has claimed that individual or group phenotypic variation and speciation primarily arise from genetic mutations and gene allelic polymorphisms (i.e., genetic drift) combined with natural selection, geographic and sexual isolation, and the interruption of gene flow between parental and emerging species. Even though this view is still going strong and favored by traditional evolutionary biologists, recent discoveries support that phenotypic variability also results from shifts of gene expression controlled by epigenetic mechanisms during ontogenesis and during adult gametogenesis. Clearly, this new information makes it possible to conceive within and across species phenotype variation and speciation as epigenetic phenomena, having no need to look for mutations as the main source of variability or to make natural selection, geographic and sexual isolation and gene flow discontinuities the forces leading to speciation (also see [1]). Variation is never made by natural selection. Natural selection acts only after the phenotypic repertoire for each species unfolds generation after generation. In the epigenetic context, phenotypic variability is an intrinsic property of individuals and arises from decisions made by the developing organism after processing and integrating the information extracted from the environment, the genome, and the metabolic state. Where and how cell decisions are made is yet unknown, but once taken they are likely to deviate ontogenetic trajectories enough to promote either the emergence of new phenotypic traits or even new species. Hence, unraveling the mechanisms underlying the epigenetic modulation of gene expression becomes central in order to understand phenotypic variation within and among species, as well as the evolutionary process of life.

 As briefly mentioned before, individual phenotypic variation is commonly “constructed” during embryonic and fetal life. It is during early ontogenetic stages when somatic cells may redirect their ontogenetic trajectories in response to epigenetic information. Commonly, this circumstance gives rise to unique, variable individuals that preserve or not, in different degrees, phenotypic features specific to the species. Hence, one of the mechanisms leading to contingent phenotypic variation is ontogenetic phenotypic plasticity of somatic cells (also called developmental phenotypic plasticity) [2].

Other critical processes that lead to ontogenetic phenotypic variability involve the epigenetic reprogramming of the genome of precursor cells that originate oocytes and spermatozoa. It is known that the first reprogramming event occurs in the gonocyte's genome (i.e., gamete primordial precursor) while colonizing the embryo's urogenital crest [3–5]. We believe this event imprints an epigenetic memory on the gonocyte's genome that “depicts” the environmental circumstances under which these cells were committed to the gamete lineage. Surely these early epigenetic memories not only influence future gamete differentiation, but the development and maturation of the organism as a whole after fertilization. A second episode of gamete epigenetic reprogramming takes place during the process of differentiation that gives rise to spermatozoa in sexually mature males. We think that by constantly reediting epigenetic memories in spermatogonial populations, this process allows spermatozoa to inherit an updated epigenome that fits current environmental circumstances. This would permit spermatozoa of different generations to provide fresh information about the environment during consecutive episodes of fertilization and to inherit this information to the offspring. Finally, a last event of epigenetic reprogramming occurs soon after fertilization. From our point of view, by mixing prenatal (mainly provided by the oocyte) and postnatal (principally provided by the spermatozoa) memories and reediting them again, based on actual environmental conditions, the zygote has a chance to create an updated epigenetic/genetic framework based on which somatic cells will take decisions to adjust the ontogenetic trajectories during prenatal and postnatal life. Hence, studying at different ages the details of the cellular and molecular underpinnings underlying epigenetic phenotypic plasticity, whether somatic or gametic, is mandatory to fully understand individuation and speciation. In doing so, the establishment of experimental models through which such details may be reasonably explored becomes critical to the field. Here is where endocrine disruptors (EDs) enter into the scene. 

EDs are a broad class of chemicals that, after modifying early or late development and maturation, promote the expression of alternative phenotypes in the exposed organisms or in their offspring. In some cases, such phenotypes are incompatible with life. In many others, however, EDs-exposed organisms display alternative adult phenotypes with variable reproductive fitness and disease susceptibility [6–8]. Even though EDs may induce mutations [9, 10], a great deal of their effects on the phenotype result from their ability to interfere with endocrine communication and/or through directly inducing epigenetic changes [9–13]. Thus, designing experiments involving the prenatal and postnatal exposure to EDs may help us understand the epigenetic bases of phenotypic variability and plasticity between individuals, across species, and throughout evolution.

In this text, we revise current knowledge about the epigenetic mechanisms that underlie the effects of EDs on phenotypic variability and plasticity. Because previous reviews have already deeply discussed EDs' epigenetic and transgenerational effects on human biology and disease, here we intend to stress the value of using the information derived from experiments with EDs to unveil the mechanisms that underlie phenotypic variability and speciation through epigenetic phenotypic plasticity.

## 2. Endocrine Disruptors: Their Chemical Nature

EDs constitute a heterogeneous group of natural and synthetic chemicals that mimic, block, or disrupt the synthesis, transport, or elimination of natural chemical messengers such as classic hormones, cytokines, and neurotransmitters [14–17]. When their active forms are released to the environment, they are absorbed by organisms through epithelial linings. Based on their physiological actions, EDs may be classified as antiandrogenic, androgenic, estrogenic, arylhydrocarbon receptor agonists, inhibitors of steroid hormone synthesis, antithyroid substances, and retinoid acid agonists [9]. Chemically, pesticides (DDT, demeton-S-methyl, dimethoate, permethrin, diazinon, and chlorfenvinphos), fungicides (vinclozolin, maneb, and metam sodium), herbicides (atrazine, simazine, linuron, diuron, and 2,4-D), industrial products (pentachlorophenol, polychlorinated biphenyls, phthalate plasticisers, alkylphenol ethoxylates, and bisphenol A), pharmaceuticals (diethylstilbestrol), nutriceuticals, and synthetic hormones used for elaborating contraceptive pills or for designing hormone replacement therapeutic schemes are among the most important EDs so far described. Also, plant and animal derived natural hormones such as phytoestrogens, 17*β*-oestradiol and testosterone may disrupt the endocrine milieu of organisms exposed to them in nature. In addition to the natural and synthetic compounds mentioned previously, it has been demonstrated that chronic hypoxia associated to organic pollution and eutrophication also exert disrupting effects on the endocrine system [18, 19]. 

## 3. Endocrine Disruptors and Phenotypic Plasticity

A number of studies conducted both in wild and in laboratory settings have convincingly shown that the prenatal exposure to EDs induces early and late onset phenotypic plasticity. For instance, prenatal exposure to the synthetic estrogen diethylstilbestrol increases the short-term risk of acquiring testicular abnormalities in men [20] and the long-term risk of developing cervical and vaginal cancer in adult women, reviewed by Rubin, [21]. Phenotypic plasticity associated with EDs exposure is not restricted, nonetheless, to prenatal developmental stages. Indeed, adult women exposed to bis4-chlorophenyl-1,1,1-trichloroethane or bis4-chlorophenyl-1,1-dichloroethene reduce or increase their fertility and develop longer or shorter than normal pregnancies, respectively [22]. Similarly, numerous cases of infertility have been reported among adult men exposed to 1,2-dibromo-3-cloropropane while working for a pesticide factory. Azoos- and oligospermia as well as increased levels of follicle-stimulating and luteinizing hormones were common findings among these men [23]. In addition, vertebrates different from humans are also affected by EDs exposure. Indeed, pregnant rats exposed to vinclozolin (an antiandrogenic compound) or methoxychlor (an estrogenic compound) during the last stages of embryonic development give rise to offspring with decreased spermatogenic capacity (cell number and viability) and increased incidence of male infertility [24].* Xenopus laevis *larvae exposed to atrazine, a commonly used herbicide, display hermaphroditism, demasculinization, and reduced testosterone plasmatic levels at adult age [25, 26]. Finally, fish exposed to oxidative stress show impaired migration of primordial germ cells [18]. Even though significant anatomical and functional differences are observed in the reproductive system of EDs-exposed organisms when compared to nonexposed ones, the emergence of alternative phenotypes is not restricted to the reproductive sphere. EDs exposure redirects the trajectory of embryonic morphogenesis and modifies also thyroid gland, immune, and neural functions during postnatal life [14, 25–29].

At this point, a consideration of the biological meaning of EDs-induced alternative phenotypes is worth doing. Although these phenotypes might be considered as “abnormalities”, from an ecological and evolutionary perspective, reducing fertility, debilitating the immune response, increasing disease susceptibility, or modifying to the organism's behavior is, however, advantageous to the species by decreasing the fitness of individuals exposed to EDs at any age. It would not make sense, for example, to permit the reproduction of exposed organisms, given their greater possibility to sire offspring that will circumstantially display maladaptive phenotypes. This is particularly significant under the light of the evidence showing that genetic expression of germ cells may be primed permanently and trans-generationally by epigenetic information during periods in which these cells undergo epigenetic programming [9, 24, 30–32]. Furthermore, recent discoveries have shown that adults exposed to EDs prenatally are less attractive to nonexposed mates [33]. Hence, at worst, these modified phenotypes must be considered as circumstantially maladaptive but never abnormal. EDs-exposed organisms might then choose from their ontogenetic alternatives the traits that better cope with EDs exposure. Therefore, the emergence of epigenetically generated seemingly maladaptive, alternative phenotypes may be a fundamental process that allows natural selection “to pick the fittest organism” at the population level under specific circumstances. 

## 4. EDs Induce Phenotypic Variability through Epigenesis

As mentioned before, EDs may act as hormonal agonists or antagonists or modify the synthesis, transport, or elimination of hormones. Hence, by changing hormone functional availability, EDs promote the expression of alternative phenotypes in developing and adult organisms. Since many of them do not induce mutations, their actions are likely translated through epigenetic mechanisms. But what does epigenesis mean? Epigenesis may be conceived as a series of cellular and molecular processes that “print out” (or encode) on to the genetic library the information extracted from the environment. This environmentally driven code restricts or facilitates the cell's access to distinct shelves of its genetic library, thus guiding the cell's search for genetic information. Once the best genetic files from the available repertoire are picked, the cell makes decisions on what ontogenetic trajectories are necessary to construct to provide a proper phenotypic response. Such processes do not involve mutations of DNA. Epigenetic information coding takes place in the genome by differentially tagging or untagging histones with acetyl, methyl, phosphoryl, ubiquitin, sumo and ADP-rybosil groups at particular amino-acid residues or the DNA with methyl groups at specific cytosine-guanine dinucleotide locations. The process of epigenetic tagging or untagging is catalyzed by enzymes (Table 1) whose activity may be modulated by different signaling cascades following the activation of receptors by their specific ligands (reviewed by Arzate-Mejía et al., [34]). The transcription of genes coding for “chromatin remodeling” enzymes may be also regulated by environmental factors [35, 36].

Chromatin epigenetic tags are either transient/removable or permanent/likely heritable. Commonly, transient/removable epigenetic tags allow the organism's cells to make moment-to-moment adjustments of their gene expression state, their metabolic status, and hence of their phenotype. Permanent epigenetic tags, in contrast, give rise to an epigenetic memory that, once posted, primes and channels each cell's adjustable genetic and metabolic responses for the rest of the organism's life. Interestingly, when permanent chromatin epigenetic tags occur in gametes, stem cells and/or amplifying precursor cells, they are inherited by their progeny both at the cellular and at the organismal level. Hence permanent epigenetic tags [37, 38], and thus past and relatively present environmental conditions, are transgenerationally heritable. Thus, the phenotype expressed by a given animal at a particular time point of life and the lifespan plasticity that such phenotype might display in response to prevailing, but changing, environmental conditions are facilitated by a highly dynamic process of epigenetic tagging channeled by the epigenetic memory. 

But how can the shifts of epigenetic tags prime and channel gene expression and metabolism in a constant and permanent manner? The trick in part lies in the stereochemistry of chromatin, whose three-dimensional structure is modified by addition and/or removal of functional chemical groups to histones and/or DNA. Chromatin relaxation or compaction lead, respectively, to the differential formation of transcriptomically active or inactive gene expression domains along the chromosomes. Also, chromatin tagging/untagging (i.e., remodeling) adjust chromosomes' nuclear topology, a circumstance that modifies gene expression by changing chromosome-chromosome spatial interactions and the spatial relationship of genes with the transcriptional factories in the cell nucleus. 

Other modes of modifying gene transcription and translation through epigenetic processes have been recently uncovered. Indeed, the insertion of histone variants, the coupling of ATP-dependent remodeling complexes and/or noncoding RNAs [39–41] also lead to chromatin remodeling. Nuclear transcription and protein synthesis may also be modified by shifting the availability of nuclear and cytoplasmic small, noncoding RNAs. Finally, genome transposable elements (e.g., transposons or retrotransposons) are now known to be regulated through epigenetic mechanisms that involve DNA methylation, interference RNAs, and hence chromatin condensation [42].

Based on the information commented, we believe EDs might use several, if not all, of the epigenetic mechanisms described to induce phenotypic plasticity. This is supported by data showing that diethylstilbestrol decreases methylation of protooncogenes and lactoferrin in mouse reproductive tissues by reducing the activity of the DNA methyl transferase-1, a condition that decreases CpG methylation [43–45]. Also, mice treated with bisphenol A either pre- or neonatally show greater body mass, modified reproductive function, increased cancer risk, and reduced DNA methylation [38, 46–48]. Similar observations have been reported in mice exposed to genistein (an estrogen-like polyphenol) [48–50], vinclozolin (a fungicide) [51], or methoxychlor (a pesticide) [24]. In fact, in the last case, alternative phenotypes may be expressed by individuals belonging to subsequent generations [24]. In rats, developmental exposure to exogenous estradiol and bisphenol A also produces permanent changes in DNA methylation levels of multiple cell signaling genes important for proper prostate development and function [47]. Another endocrine disrupting compound, the insecticide methoxychlor, was found to modify DNA methylation patterns of the rat germ cell line when administered during development. It also decreases sperm number and viability and causes infertility across generations [24]. In male rats, vinclozoline modifies both the testis transcriptome and epigenome transgenerationally through modifying DNA methylation during development [7, 30, 51]. Vinclozoline also shifts sperm methylation levels of at least six known imprinted genes throughout three generations [52]. Using a reporter gene H19, it was recently found that the pesticide chlorpyrifos affects DNA methylation patterns in male mice primordial germ and liver cells [53]. A very recent study in mice has revealed that gestational exposure with the dioxin 2,3,7,8-Tetrachlorodibenzo-*p*-dioxin shifts interference RNA availability and DNA methylation patterns in the offspring of exposed females [54]. More recent studies have shown that the exposure of steroidogenic tissues to gonadotropins in male and female mice induces the expression of VL30 retrotransposons [55]. Also, benzo(a)pyrene exposure induces the trimethylation of the lysine 4 residue and the acetylation of the lysine 9 residue of histone 3 leading to the downregulation of the expression of the DNA methyltransferase 1 locus and the upregulation of the LINE-1 retrotransposon site [56]. Finally, pregnant mice exposed to bisphenol A show hypomethylation of an intracisternal A-particle retrotransposon located upstream of the Agouti gene. This effect was counteracted by supplementing maternal diets with methyl donors [38]. These last results support that EDs may also exert their action on phenotypic plasticity by promoting mobilizations of these elements. Transposons and retrotransposons are replicative DNA sequences that can move across chromosomes [57, 58]. The transposition of these elements among chomosomes is achieved after having them cleaved, transcribed, or retrotranscribed. Transcription, retrotranscription, cleavage, and transposition are all mediated by distinct families of enzymes and a host of interference RNAs that work in an orchestrated fashion [58, 59]. 

## 5. EDs as Tools to Explore the Evolution of Life

**Figure 1:**
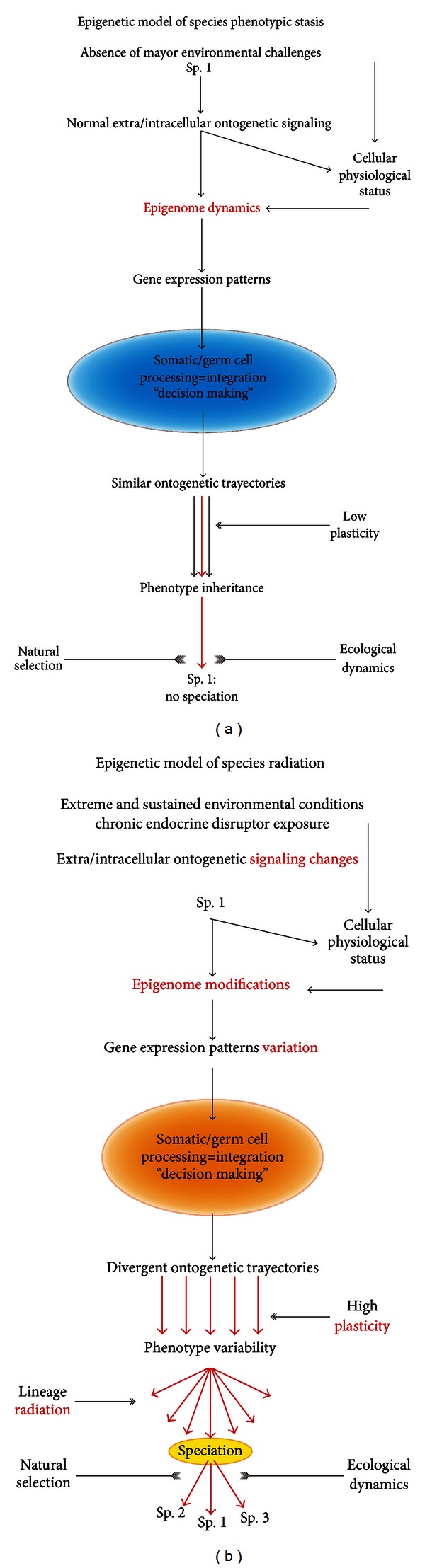
Diagrams that illustrate the hypothetical models by which the phenotype of a given animal species may be preserved under normal environmental conditions (a) or diversified under extreme environmental conditions (b). Phenotypic stasis (a) or speciation (b), respectively, are the end results of these models. Sp., Species.

Based on what we have written so far, we hope that the reader concurs with the idea that the variations of the phenotype within and across species achieved through epigenetic phenotypic plasticity might be a driving force of phenotypic variability and perhaps of speciation. Although many may argue against the value of using EDs exposure to understand speciation since they promote the emergence of seemingly maladaptive phenotypes with reduced fitness, we must remember that events of speciation (e.g., adaptive radiation) may occur following massive extinctions induced by climatic catastrophes [60]. Such circumstances surely expose the surviving organisms to extreme environmental conditions that likely force them to develop extreme phenotypic plasticity to thrive over time. EDs exposed organisms might display phenotypes that resemble those shown by organisms exposed to extreme climatic challenges [61]. It is known that highly stressful conditions impair somewhat reproductive fitness and may lead to phenotypic “abnormalities.” However, these “abnormalities” might be the raw substrate upon which extreme phenotypic plasticity may be built up giving rise to new species traits. In this scenario, EDs could help us understand how epigenomes are configured under such extreme circumstances and how they influence the decisions taken by developing organisms to select their ontogenetic trajectories. In this process, the mobility and overexpression of transposable elements induced by EDs exposure may be particularly important since they could modify developmental processes as important as body patterning [62], see also [63]. Because such actions result from the interpretation of the epigenomic code by the somatic and germ cells of the developing system, such changes may be transgenerationally inherited. How far these changes may last is unclear, but given the dynamic nature of epigenomes, they may be perpetuated or reedited based on the environmental conditions as they evolve. Hence, EDs could generate a phenotypic variation by reconfiguring the epigenome that could lead to divergent phenotypes based upon changes of gene expression patterns. In addition, EDs could also enhance phenotypic plasticity by promoting novel DNA recombination events after increasing the mobility of transposable elements through epigenetic modulation. We believe that both processes might lead in the long run to *epigenetic species radiation* without point mutations (Figure 1). Our arguments fully concord with the philosophical framework posed by the emerging field of environmental epigenetics [1].

## Figures and Tables

**Table 1:** Enzymes involved in chromatin epigenetic tagging/untagging [2–4].

Epigenetic modification (tagging/untagging)	Enzymes
*DNA modifications: *	*DNA modifying enzymes *
Methylation	DNA methyltransferases (DNMTs)
*Histone modifications: *	*Histone modifying enzymes *
Acetylation of specific lysine residues/Deacetylation	Histone acetyltransferases (HATs)/Histone deacetylases (HDACs)
Methylation of specific lysine or arginine residues/Demethylation	Histone methyltransferases (HKMTs and HRMTs)/Lysine-specific demethylase (LSD1); arginine-deiminases
Phosphorylation of serine or threonine groups/dephosphorylation	Histone Kinases (HKs)/Phosphatases (PPTases)
Ubiquitinylation/removal of ubiquitin	Ubiquitinases/“deubiquitinases” or ubiquitin hydrolases (Ubps)
Sumoylation	SUMO E3 ligase
Poly(ADP-ribosyl)ation/removal of Poly(ADP-ribose) units	PAR polymerases/poly(ADP-ribose)glycohydrolases (PARGs)
